# GATA factor genes in the *Drosophila* midgut embryo

**DOI:** 10.1371/journal.pone.0193612

**Published:** 2018-03-08

**Authors:** Beatriz Hernández de Madrid, Jordi Casanova

**Affiliations:** 1 Institut de Biologia Molecular de Barcelona (IBMB-CSIC), Barcelona, Catalonia, Spain; 2 Institute for Research in Biomedicine (IRB Barcelona), The Barcelona Institute of Science and Technology (BIST), Barcelona, Catalonia, Spain; University of Dayton, UNITED STATES

## Abstract

The Drosophila GATA factor gene *serpent* (*srp*) is required for the early differentiation of the anterior and posterior midgut primordia. In particular, *srp* is sufficient and necessary for the primordial gut cells to undertake an epithelial-to-mesenchimal transition (EMT). Two other GATA factor genes, *dGATAe* and *grain* (*grn*), are also specifically expressed in the midgut. On the one hand, d*GATAe* expression is activated by *srp*. Embryos homozygous for a deficiency uncovering *dGATAe* were shown to lack the expression of some differentiated midgut genes. Moreover, ectopic expression of *dGATAe* was sufficient to drive the expression of some of these differentiation marker genes, thus establishing the role of *dGATAe* in the regulation of their expression. However, due to the gross abnormalities associated with this deficiency, it was not possible to assess whether, similarly to *srp*, *dGATAe* might play a role in setting the midgut morphology. To further investigate this role we decided to generate a *dGATAe* mutant. On the other hand, *grn* is expressed in the midgut primordia around stage 11 and remains expressed until the end of embryogenesis. Yet, no midgut function has been described for *grn*. First, here we report that, as for *dGATAe*, midgut *grn* expression is dependent on *srp*; conversely, *dGATAe* and *grn* expression are independent of each other. Our results also indicate that, unlike *srp*, *dGATAe* and *grn* are not responsible for setting the general embryonic midgut morphology. We also show that the analysed midgut genes whose expression is lacking in embryos homozygous for a deficiency uncovering *dGATAe* are indeed *dGATAe*-dependent genes. Conversely, we do not find any midgut gene to be *grn*-dependent, with the exception of midgut repression of the proventriculus *iroquois* (*iro*) gene. In conclusion, our results clarify the expression patterns and function of the GATA factor genes expressed in the embryonic midgut.

## Introduction

The GATA proteins are a family of transcription factors that regulate diverse genetic programs during development. In vertebrates, the six GATAs can be comprised in two subfamilies: GATA-1/2/3 and GATA-4/5/6. Members of the first group play important roles in the hematopoietic system, while the second group is mainly expressed in endodermal tissues and their miss regulation has been associated with gastrointestinal malignancy such as those of the stomach, pancreas and colon [1,2]. Likewise, in Drosophila GATA factors have both a role in the blood cell lineage and in endoderm cells. Among the Drosophila GATA factor genes, *serpent* (*srp*) is required for the early differentiation of endodermal cells in the anterior and posterior midgut primordia [3]. In particular, expression of *srp* is sufficient and necessary for these epithelial cells to undertake an epithelial-to-mesenchimal transition (EMT), a feature shared by the human GATA-6, which induces a similar transition in mammalian cells [4]. By embryonic stage 10, once the midgut cells initiate migration, *srp* expression decays.

Two other GATA factor genes, *dGATAe* and *grain* (*grn*), are also specifically expressed in the midgut, partially overlapping with *srp* expression and extending to later stages of development. On the one hand, d*GATAe* is first detected at stage 8 in the endoderm, and its expression is activated by *srp* [5]. Embryos homozygous for a deficiency uncovering *dGATAe* as well as at least other 12 genes were shown to lack the expression of some genes used as markers of the differentiated midgut. Moreover, ectopic expression of *dGATAe* was sufficient to drive the expression of some of these differentiation marker genes, thus establishing the role of *dGATAe* in the regulation of their expression [5,6]. However, due to the gross abnormalities associated with this deficiency, it was not possible to assess whether, similarly to *srp*, *dGATAe* might play a role in setting the midgut morphology. On the other hand, *grn* is expressed in the endoderm around stage 11 and it remains expressed there until the end of embryogenesis. Yet, no specific endodermal function has been described for *grn*.

To further investigate the role of GATA factor genes in the embryonic gut morphogenesis we decided to generate a *dGATAe* mutant. We then used the newly induced *dGATAe* mutant as well as an already available *grn* mutant to extend the previous results and establish the functional relationship between the midgut *GATA* genes, and between them and the genes expressed in the differentiated embryonic midgut.

## Results and discussion

### Generation of a *dGATAe* mutant

**Fig 1:**
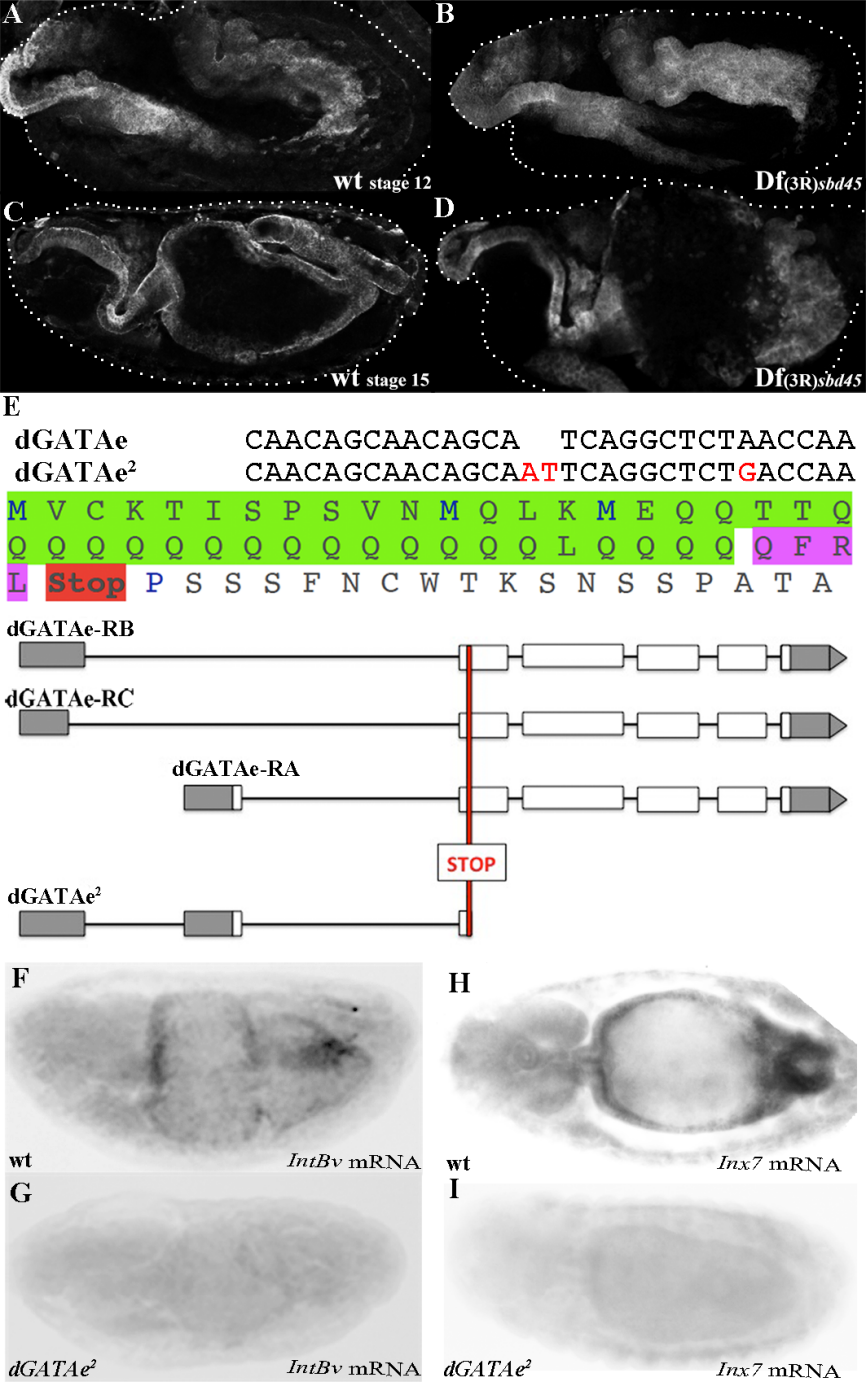
Generation of a *dGATAe* mutant. **(A-D)** Wild type and Df(3R)*sbd*^*45*^ embryos at stages 12 and 15. In the wild-type (A,C) the anterior and posterior midgut primordia reach the embryonic central region and fuse to develop the midgut while Df(3R) *sbd*^*45*^ embryos (B,D) show gross abnormalities; the midgut primordia are visualised by GFP expressed under the control of a *hkb*-GAL4 construct. The dotted lines indicate the contour of the embryos. **(E)** Wild-type DNA *dGATAe* sequence and the CRISPR-induced change in the *dGATAe*^*2*^. The change introduces a very early stop codon in the dGATAe protein from any of the identified RNA messages. **(F,G)** *intβν* mRNA accumulates in the midgut of the wild-type embryos but is absent in *dGATAe*^*2*^ homozygous embryos. **(H,I)** *inx7* mRNA accumulates in the midgut of the wild-type embryos but is absent in *dGATAe*^*2*^ homozygous embryos.

As mentioned above, previous studies on the role of *dGATAe* in the embryo relied on the Df(3R)*sbd*^*45*^, a deficiency uncovering *dGATAe*, as well as at least other 12 genes [5]. However, embryos homozygous for this deficiency show such gross morphological abnormalities, already starting at stage 12, that it was not possible to ascertain a role for *dGATAe* in midgut morphogenesis based on this deficiency (Fig 1B and 1D). At the time of starting this project there were no available mutants for *dGATAe* and thus we decided to generate a null allele with CRISPR (see materials and methods and Fig 1E). The resulting mutant gene is predicted to produce a dGATAe truncated protein of only 45 amino acids instead of the native 746. Accordingly, homozygous embryos for this mutation lack expression of two previously identified *dGATAe*-dependent genes, *integrinβν* (*intβν*) and *innexin7* (*inx7*) [6] (see below) (Fig 1G and 1I). While undergoing this work, the Adachi-Yamada’s group generated another *dGATAe* mutant that lacks almost all the coding region [7]. This mutation, named *dGATAe*^*1*^, failed to complement the mutation induced in our laboratory, that hence we named *dGATAe*^*2*^.

### Functional relationship between *srp*, *dGATAe* and *grn*

**Fig 2:**
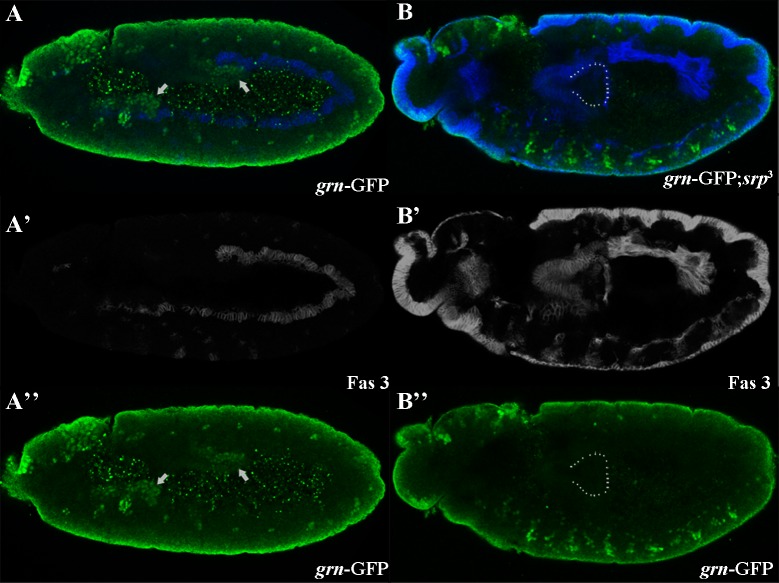
Endoderm *grn* expression is downstream of *srp*. (A,B) At germ band extension, *grn* (in green) accumulates at the anterior and at the posterior midgut in wild-type embryos (arrows in A) as detected with a GFP insertion in the endogenous *grn* gene. Conversely, it is absent in the corresponding cells in *srp* mutants (dotted line for the posterior cells), which do not develop into a midgut. Fas3 (in blue) labels the visceral mesoderm. A',A",B' and B" show the corresponding images in the blue and green channels respectively.

**Fig 3:**
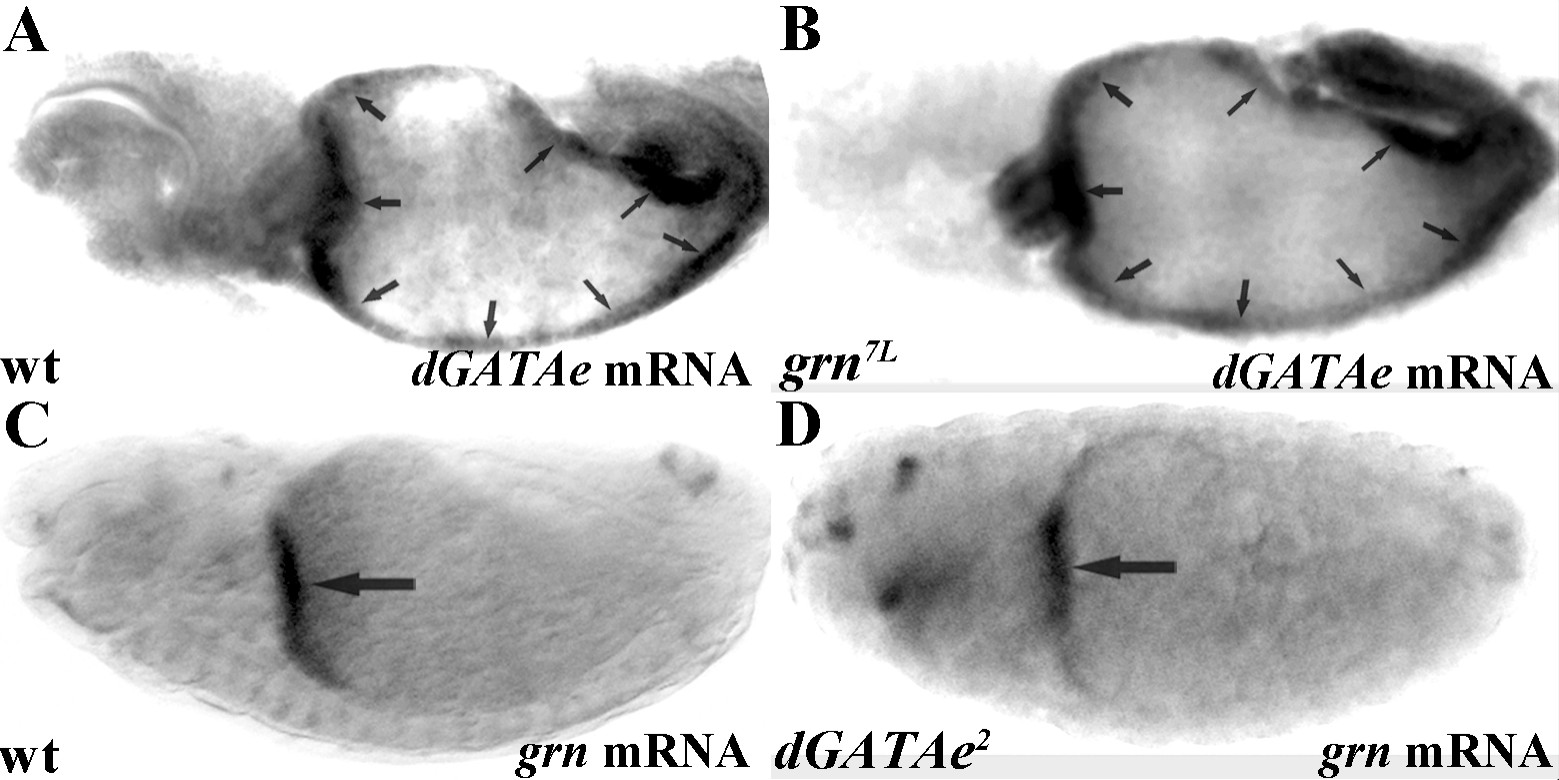
Midgut expression of *dGATAe* and *grn* are mutually independent. (A,B) *dGATAe* transcripts accumulate at the midgut of wild-type embryos (A) as well as at the midgut of *grn* mutants (arrows) (B). (C,D) Similarly, *grn* mRNA accumulates at the anterior midgut but it has faded away from the posterior midgut in wild-type embryos (C); the same pattern is detected in *dGATAe* mutant embryos (D). *dGATAe* and *grn* expression are detected by in situ hybridisation.

As previously indicated, *dGATAe* expression is downstream of *srp* [5]. We also found this to be the case for *grn* as its RNA is not detected at the endoderm of *srp* mutant embryos (see S1 Fig); we have corroborated it by means of a GFP insertion on the endogenous *grn* gene (see materials and methods) (Fig 2B). We also found *dGATAe* and *grn* expression to be independent of each other as revealed by the expression of each of the two genes in mutant embryos for the other gene (Fig 3B and 3D). Then we analysed whether *grn* and *dGATAe* might be part of a feedback loop mechanism to regulate *srp* expression, since *srp* expression decays at the onset of *dGATAe* and *grn* expression. But this is not the case; because of the overlap between *dGATAe* and *grn* expression and to discard any redundancy we analysed *srp* expression in embryos double mutant for both d*GATAe* and *grn* and found *srp* normally decaying (S2 Fig).

### Role of *dGATAe* and *grn* in the development of the embryonic midgut

We did not detect morphological abnormalities in the midgut of mutant embryos for either *dGATAe* or *grn* or mutant for both *dGATAe* and *grn* (S3 Fig). We then assessed the role of *dGATAe* and *grn* in the regulation of the midgut genes known to depend on *srp* expression.

First, we choose two *srp*-dependent genes whose expression is absent in the Df(3R)*sbd*^*45*^ homozygous embryos [5] and by analysing their expression in *dGATAe*^*2*^ mutants have shown that they did depend on *dGATAe* function (Fig 1F–1I). These observations clearly support the initial proposition that the *srp*-dependent genes whose expression is absent in the Df(3R)*sbd*^*45*^ homozygous embryos are indeed *dGATAe* dependent [5,6].

**Fig 4:**
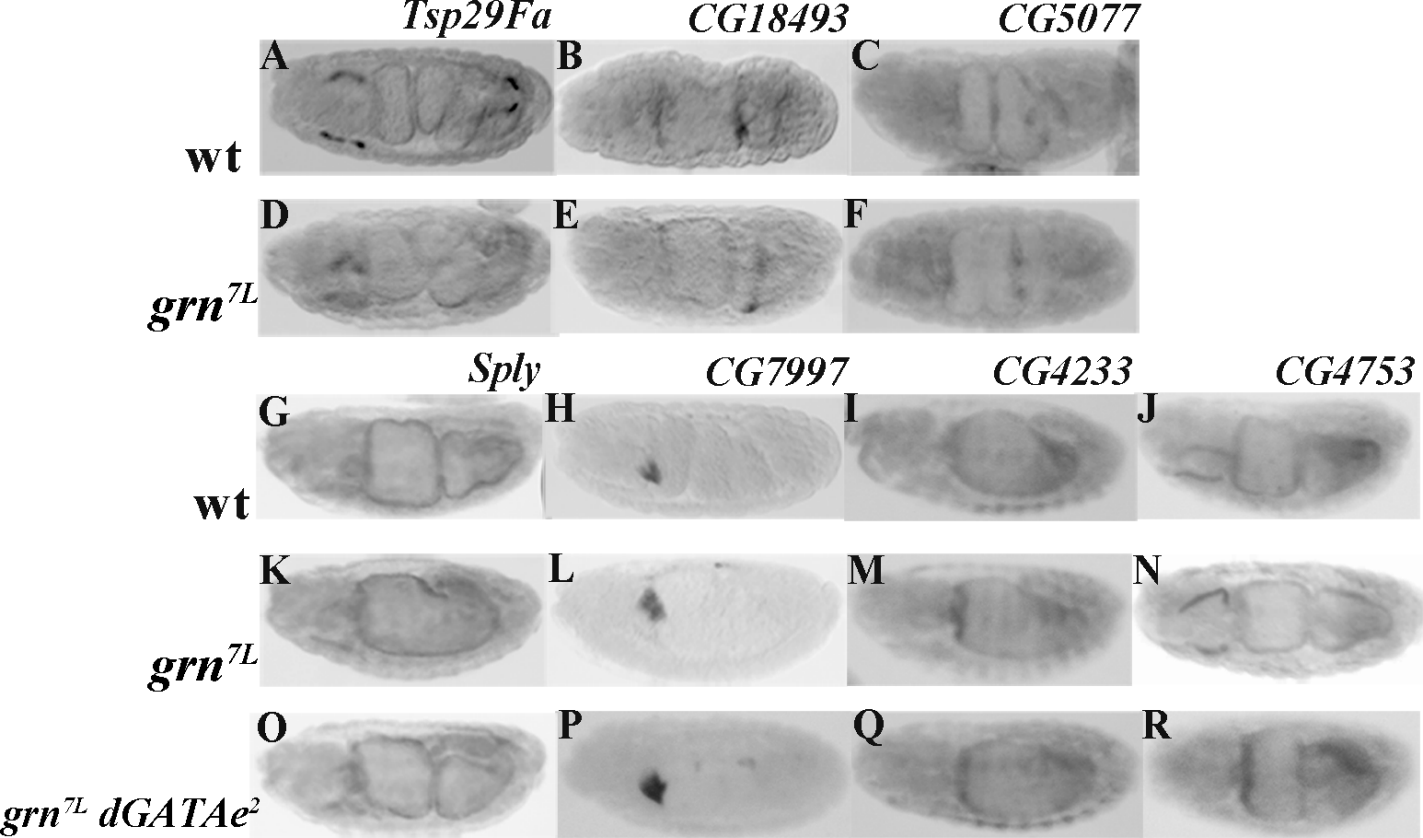
Expression of midgut genes in *grn* and in *grn dGATAe* double mutants. (A-C) Midgut genes for which *dGATAe* is necessary but not sufficient for their expression; these genes are normally expressed in *grn* mutants (D-F). (G-J) Midgut expression of genes for which *srp*, but not *dGATAe*, is required for their expression; these genes are normally expressed in grn mutants (K-N) and in embryos doubly mutant for both *grn* and *dGATAe* (O-R). Gene expression is detected by in situ hybridisation.

Second, we analysed the role of *grn* in the regulation of midgut gene expression. To this end we split the *srp*-dependent genes in 4 groups according to the data from the Okumura laboratory [6]. A first group comprises the genes that depend on *dGATAe* expression and which have a generalized expression upon ectopic expression of dGATAe (*intβν*, *inx7*, *mex1*, CG4781, CG10300, λ*Trypsin*); indeed, this generalized expression is much broader that the normal domain of *grn* expression suggesting that this six genes do not depend on *grn* and that *dGATAe* is both necessary and sufficient for their expression. A second group comprises the genes for which *dGATAe* is necessary but not sufficient as they are not ectopically expressed upon *dGATAe* ectopic expression (*Tsp29Fa*, CG18493, CG5077); we found that these genes do not require *grn* as they are normally expressed in *grn* mutant embryos (Fig 4D–4F). A third group comprises the genes neither requiring *dGATAe* for their wild-type expression nor being activated outside their normal location upon *dGATAe* ectopic expression (*Sply*, CG7997, CG4233); these are indeed the better candidates for genes depending on *grn* activity for their expression but we found them to be normally expressed in *grn* mutants (Fig 4K–4M). To discard a possible redundancy between *grn* and *dGATAe* in promoting the expression of these genes we also analysed their expression in embryos doubly mutant for both *grn* and *dGATAe* and found them to be normally expressed in these embryos as well (Fig 4O–4Q). Finally, a fourth group is comprised by genes that do not require *dGATAe* for their wild-type expression but are activated outside their normal location upon d*GATAe* ectopic expression (*hnf4*, CG4753); this kind of genes are also normally expressed both in *grn* mutant and in *grn dGATAe* double mutant embryos (Fig 4N and 4R and S4 Fig). In sum, none of the srp-dependent genes identified so far in the embryonic midgut appears to be grn-dependent.

**Fig 5:**
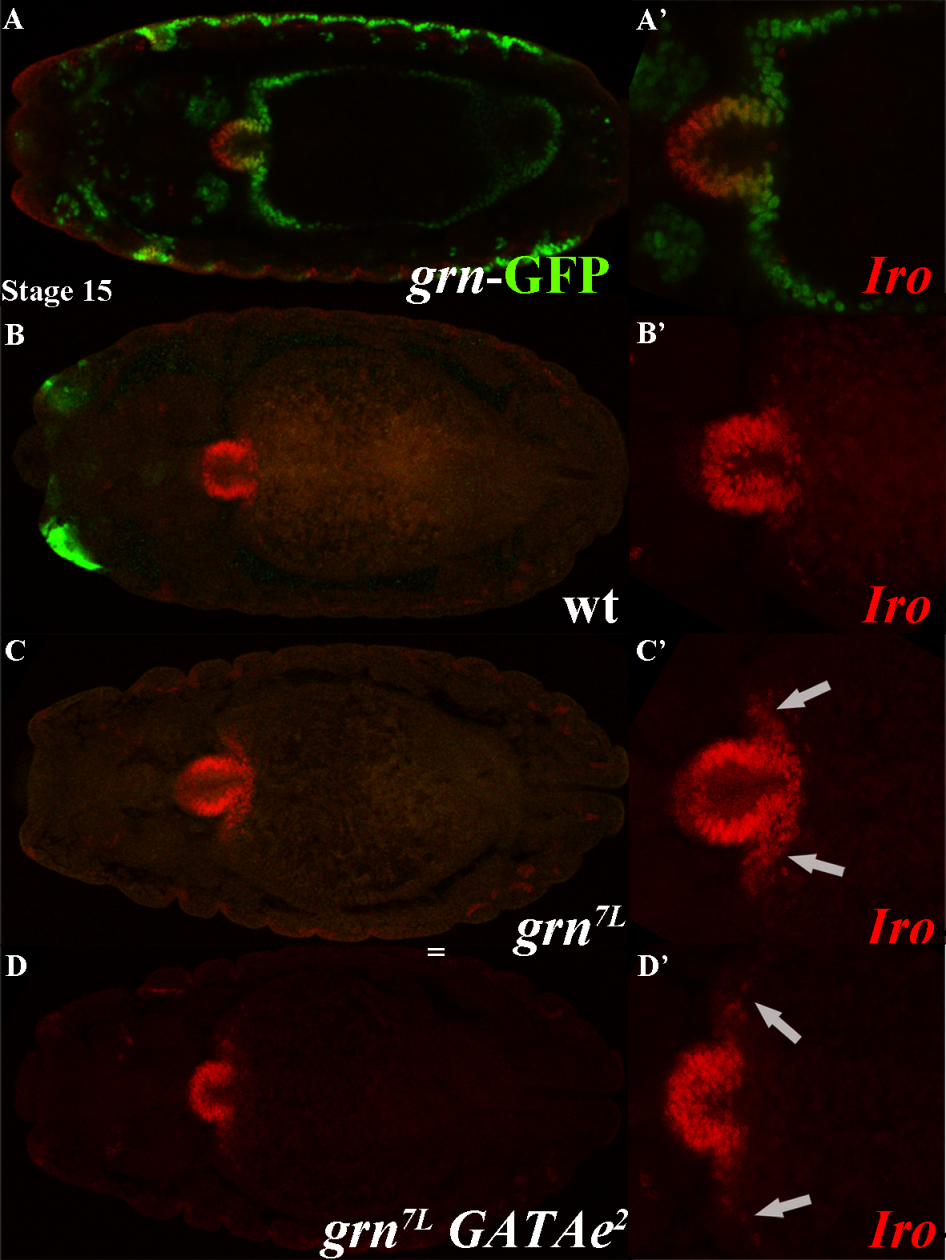
*grn* and iro expression at the anterior midgut. (A) Partial overlap between *grn* and iro expression at the anterior midgut; (A´) magnification of the anterior midgut region. (B) Same image as in A only in the red channel to visualise *iro* expression; (B´) magnification of the anterior midgut region. (C) iro expression expands in the anterior midgut of *grn* mutants; (C´) magnification of the anterior midgut region. (D) expression of iro is not further expanded in mutant embryos for both *dGATAe* and *grn;* (D´) magnification of the anterior midgut region. *grn* and iro expression are detected by means of a GFP insertion on the endogenous *grn* gene (in green) and an anti-iro antibody (in red). Arrows in C and D' indicate the expansion of iro into the anterior midgut.

Third, we paid a special attention to the proventriculus, the structure at the junction between the foregut and the midgut that has been shown to be defective in the *dGATAe* mutant larvae [7]. In particular, we examined the expression of many genes with specific expression patterns in the proventriculus [*forkhead* (*fkh*), *short stop* (*shot*), *wingless* (*wg*), *drumstick* (*drm*), *bowl* (*bowl*) and *iroquois* (*iro*)] and found no change in *dGATAe* mutants (for expression of *fkh*, *wg* and *shot* see S5 Fig). Nevertheless, we found a partial overlap between *grn* and *iro* expression (Fig 5A) and moreover that *grn* acts as a repressor of *iro* at the anterior part of the midgut as *iro* expression expands in *grn* mutant embryos (Fig 5C); expression of *iro* is not further expanded in mutant embryos for both *dGATAe* and *grn* (Fig 5D).

**Fig 6:**
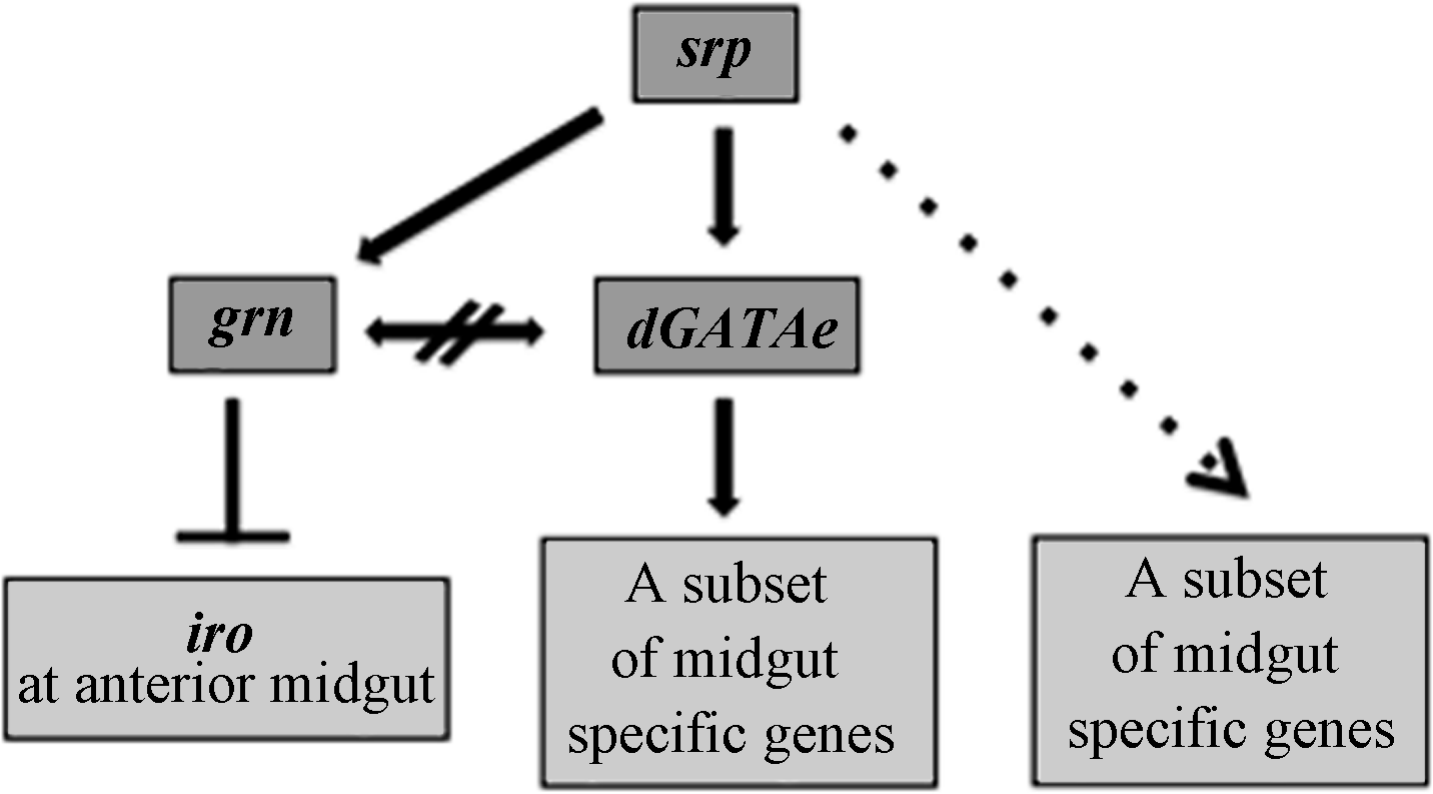
A model for the regulation of midgut gene expression. Regulatory interactions among the genes analysed in this work. The dotted line indicates that it is not possible to rule out an intermediate step similar to the one for the other differentiation genes. The cross out double arrow indicates *dGATAe* and *grn* expression being independent of each other.

In conclusion, our results clarify the expression patterns and function of the GATA factor encoding genes expressed specifically in the embryonic midgut (Fig 6). We show that both *dGATAe* and *grn* are independent *srp* target genes that do not regulate each other. *dGATAe*, while regulating many specific midgut genes, is not involved in setting the morphology of the embryonic midgut. Some of the genes regulated by *dGATAe* have been claimed to be terminal differentiation genes [5]. The lack of a gut morphological phenotype in *dGATAe* mutant embryos suggests that these terminal differentiation genes are more likely involved in physiological functions rather than in morphological processes. However, this is not always the case. Thus, for example, *dGATAe* regulates the expression of the gene encoding the *β*νintegrin subunit, which has a role in midgut cell migration; however, this role can only be unveiled in the absence of the *β*PS integrin subunit [8], thus accounting for the lack of a migration phenotype in the *dGATAe* mutant embryos. Conversely, we have found a very limited role for *grn* in the embryonic gut, only in restricting *iro* expression in its more anterior region. In addition, our results also indicate that while both *dGATAe* and *grn* are GATA proteins that accumulate in overlapping domains, they do not appear to show any redundant function.

## Materials and methods

### *Drosophila* stocks

The following strains were used: *dGATAe*^*1*^, *dGATAe*^*2*^, *grn*^7L^, PBac{grn-GFP.FPTB}VK00037, the recombinant *grn*^7L^ *dGATAe*^*1*^, Deficiency Df(3R)*sbd*^*45*^, *hkb-*GAL4 and UAS-GFP. Details for genotypes and transgenes can be found in flybase (http://flybase.org). *dGATAe*^*1*^ was kindly provided by Takashi Okumura. Other stocks were obtained from the Bloomington Stock Center.

### *Drosophila* genetics and transgenic lines

The *dGATAe*^*2*^ allele was generated by CRISPR-Cas9-mediated editing. A guide RNA [(gRNA) GTCGATTGCAACAGCAACAGCATCGTT] was designed to target the first common exon of the three Drosophila *dGATAe* isoforms. The gRNA construct was prepared in the vector pCDF3 [9] and inserted at the attP40 landing site via phiC31-mediated integration [10]. Transgenic gRNA males were crossed to nanos-cas9 females to obtain founder males, which were then crossed to females carrying the TM3 balancer for recovery of mutant alleles. Induced mutations were characterized by sequencing PCR fragments amplified from candidate flies.

### *In situ* hybridization

*In situ* hybridization was performed using a standard protocol [11]. Digoxigenin–UTP-labelled antisense RNA probes for *IntΒυ*, *Tsp29Fa*, *CG18493*, *CG5077*, *Sply*, *CG7997*, *CG4233*, *CG4753*,*Bowl*, *Drum*, *dGATAe* and *grn* were prepared from genomic DNA.

### Immunostaining

Embryos were fixed, mounted, and staged using standard techniques. Immunostaining was performed using standard protocols. Embryos were fixed in 4% formaldehyde-PBS-heptane, using standard techniques. Primary incubations were performed overnight, followed by incubation with appropriate secondary antibodies. Images were taken using standard confocal microscopy (Leica SPE) and post-processed with Adobe Photoshop and ImageJ. The following antibodies were used: goat anti-GFP Rockland), mouse anti-Fas3 (Developmental Studies Hybridoma Bank), guinea pig anti-Fkh (gift from Pilar Carrera) mouse anti-Wg (Developmental Studies Hybridoma Bank), mouse anti-Shot (Developmental Studies Hybridoma Bank), rabbit anti-Iro (gift from Mar Ruiz), rat anti-Srp (from our own lab) rat anti-Hnf4 (gift from A. Casali). Secondary antibodies were anti-goat Cy2, anti-rabbit Cy3, and anti-mouse-Cy5 at 1/150 (Jackson ImmunoResearch).

## Supporting information

S1 Fig *grn* expression. *grn* expression as assessed by *in situ* hybridisation in wild type (A,C,E) and *srp* mutant embryos (B,D,F) at stage 12 (A,B), stage 13 (C,D) and stage 14 (E,F). *grn* is expressed in the anterior and posterior midgut in the wild type (A,C,E) but is absent in the midgut (red dotted lines) in *srp* mutants (B,D,F). *grn* is also detected in structures of the ectoderm, such as the posterior spiracles, in both wild type and *srp* mutant embryos.
(PSD)

S2 Fig Midgut accumulation of srp protein. By stage 12, Srp protein can be detected by an anti-srp antibody in the migrating posterior midgut (dotted line) both in wild-type and in *grn dGATAe* double mutant embryos. However, by stage 13 we do not detect Srp in the midgut of either wild-type or *grn dGATAe* double mutant embryos (dotted midline).
(JPG)

S3 Fig Midgut morphology. Wild type and *grn dGATAe* double mutant embryos stained with Fasciclin 3 to mark the visceral muscle. In *grn dGATAe* double mutant embryos the three gut constrictions are perfectly formed and the shape of the gut is not different from the wild type one.
(TIF)

S4 Fig Midgut accumulation of Hnf-4 protein. Wild type and *grn* mutant embryos at stage 13 show the same pattern of Hnf-4 midgut accumulation as detected by antibody staining (arrows).
(TIF)

S5 Fig Accumulation of proventriculus proteins. No differences are observed in the proventriculus accumulation of either Fkh, Wg or Shot as detected by antibodies in wild type, *grn* or *dGATAe* mutant stage 16 embryos. The proventriculus is a rapidly evolving structure that is not easy to reproduce in a two-dimensional figure and thus some images may look a bit different.
(JPG)
