# Supplementary figures and images for: GATA factor genes in the Drosophila midgut embryo
Source: PLoS One. 2018 Mar 8;13(3):e0193612. doi: 10.1371/journal.pone.0193612 (PMC5843246; doi:10.1371/journal.pone.0193612)

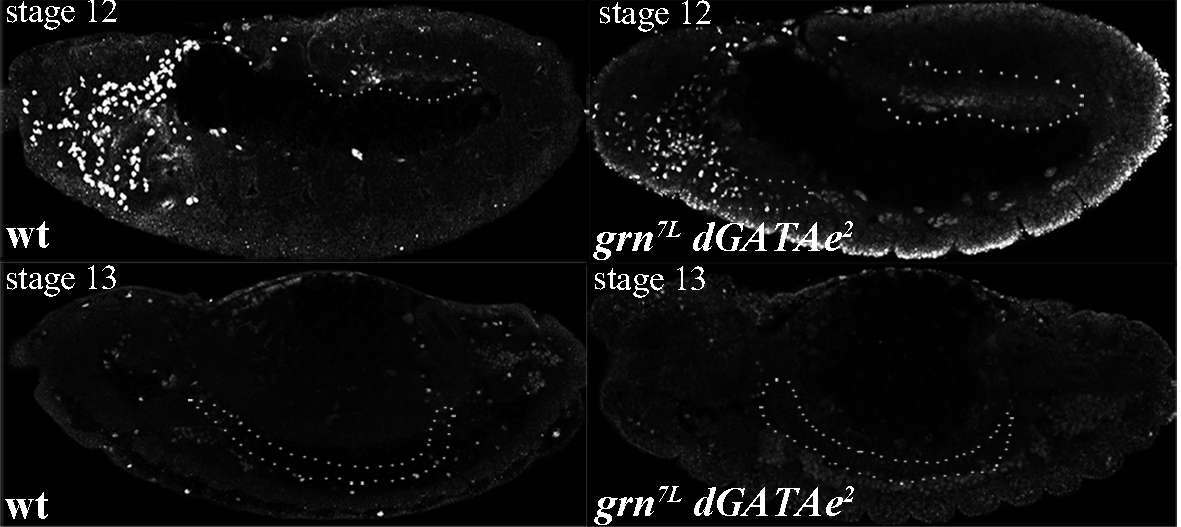

Supplement: S2 Fig — By stage 12, Srp protein can be detected by an anti-srp antibody in the migrating posterior midgut (dotted line) both in wild-type and in grn dGATAe double mutant embryos. However, by stage 13 we do not detect Srp in the midgut of either wild-type or grn dGATAe double mutant embryos (dotted midline). (JPG) [file pone.0193612.s002.jpg]

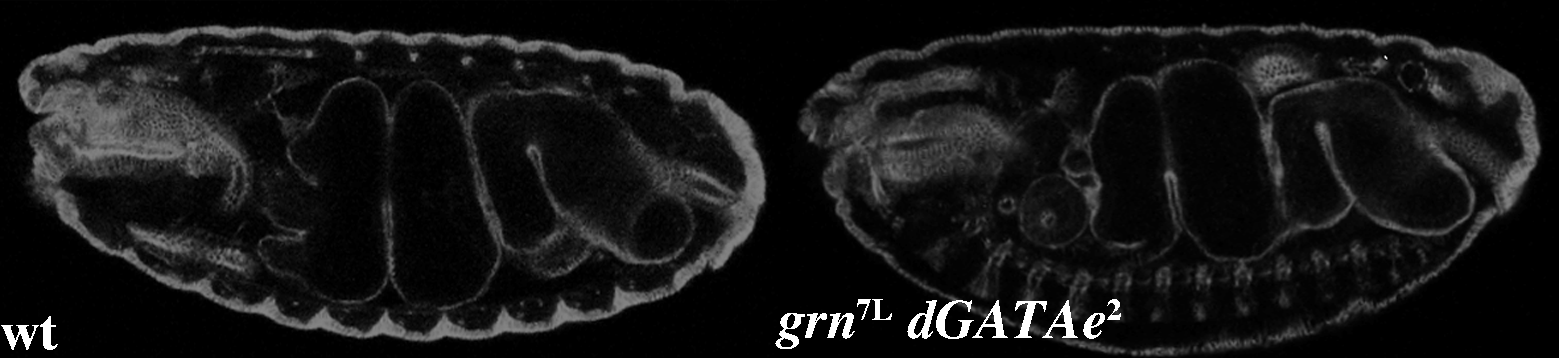

Supplement: S3 Fig — Wild type and grn dGATAe double mutant embryos stained with Fasciclin 3 to mark the visceral muscle. In grn dGATAe double mutant embryos the three gut constrictions are perfectly formed and the shape of the gut is not different from the wild type one. (TIF) [file pone.0193612.s003.tif]

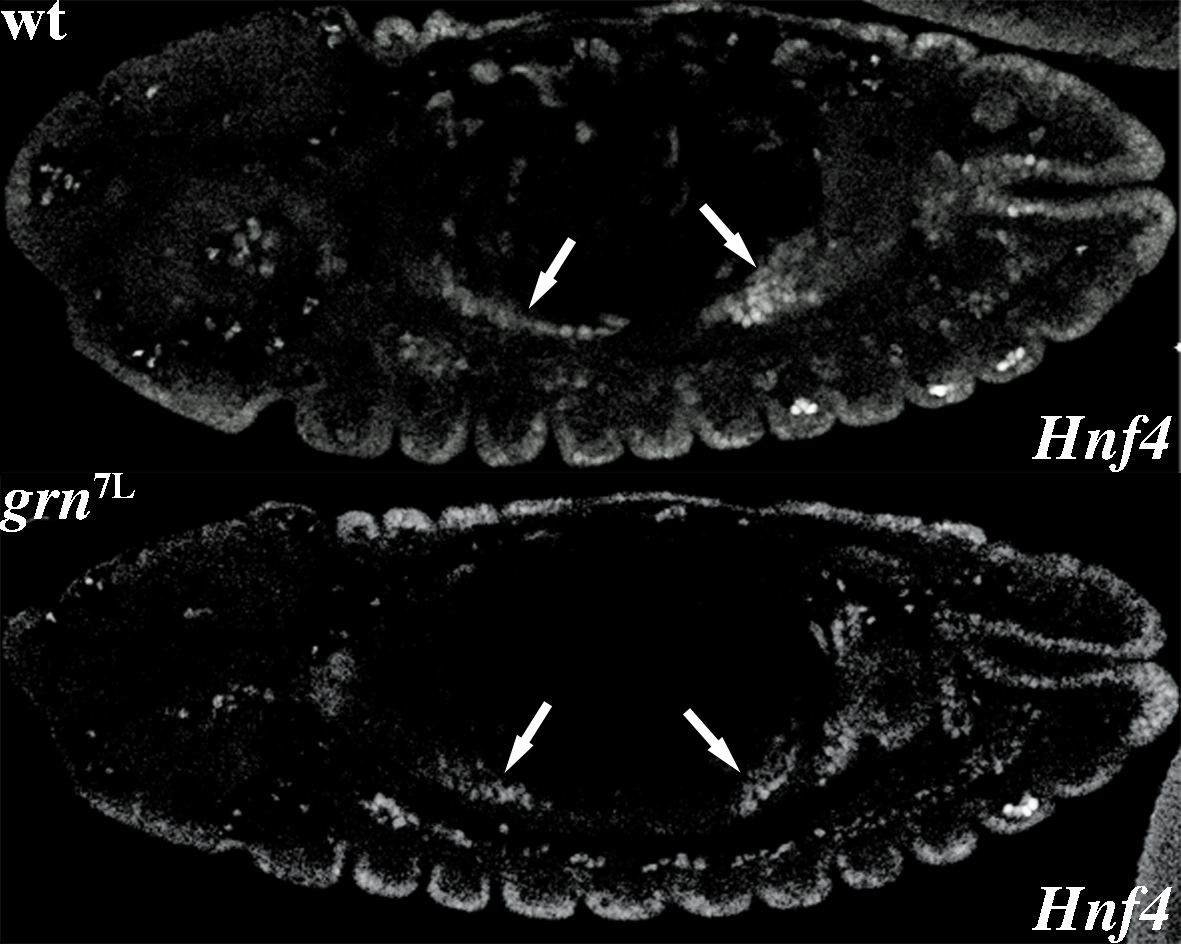

Supplement: S4 Fig — Wild type and grn mutant embryos at stage 13 show the same pattern of Hnf-4 midgut accumulation as detected by antibody staining (arrows). (TIF) [file pone.0193612.s004.tif]
